# NAA10 dysfunction with normal NatA-complex activity in a girl with non-syndromic ID and a de novo *NAA10* p.(V111G) variant – a case report

**DOI:** 10.1186/s12881-018-0559-z

**Published:** 2018-03-20

**Authors:** Nina McTiernan, Svein Isungset Støve, Ingvild Aukrust, Marita Torrisen Mårli, Line M. Myklebust, Gunnar Houge, Thomas Arnesen

**Affiliations:** 1 Department of Biological Sciences, University of Bergen, Bergen, Norway; 2 Department of Biomedicine, University of Bergen, Jonas Lies vei 91, N-5020 Bergen, Norway; 3 Department of Medical Genetics, Haukeland University Hospital, N-5021 Bergen, Norway; 4 Department of Surgery, Haukeland University Hospital, Bergen, Norway

**Keywords:** NAA10, X-linked, XLID, Developmental delay, Intellectual disability, N-alpha-acetyltransferase, Acetylation, NatA

## Abstract

**Background:**

The NAA10-NAA15 (NatA) protein complex is an N-terminal acetyltransferase responsible for acetylating ~ 40% of eukaryotic proteins. In recent years, *NAA10* variants have been found in patients with an X-linked developmental disorder called Ogden syndrome in its most severe form and, in other familial or de novo cases, with variable degrees of syndromic intellectual disability (ID) affecting both sexes.

**Case presentation:**

Here we report and functionally characterize a novel and de novo *NAA10* (NM_003491.3) c.332 T > G p.(V111G) missense variant, that was detected by trio-based whole exome sequencing in an 11 year old girl with mild/moderate non-syndromic intellectual disability. She had delayed motor and language development, but normal behavior without autistic traits. Her blood leukocyte X-inactivation pattern was within normal range (80/20). Functional characterization of NAA10-V111G by cycloheximide chase experiments suggests that NAA10-V111G has a reduced stability compared to NAA10-WT, and in vitro acetylation assays revealed a reduced enzymatic activity of monomeric NAA10-V111G but not for NAA10-V111G in complex with NAA15 (NatA enzymatic activity).

**Conclusions:**

We show that NAA10-V111G has a reduced stability and monomeric catalytic activity, while NatA function remains unaltered. This is the first example of isolated NAA10 dysfunction in a case of ID, suggesting that the syndromic cases may also require a degree of compromised NatA function.

## Background

The NAA10-NAA15 protein complex (NatA) is an N-terminal acetyltransferase (NAT) responsible for acetylating (Nt-acetylating) ~ 40% of eukaryotic proteins [1, 2]. NAA10 is the catalytic subunit that NAA15 is docking to the ribosome, forming a catalytically active complex (the NatA complex) that acetylates N-termini of newly synthesized peptide chains as the chain emerges from the ribosomal exit tunnel [3–7]. The NatA complex will acetylate peptide chains with either serine, alanine, glycine, threonine, valine or cysteine as the N-terminal residue after the initiator methionine has been cleaved off by Methionine aminopeptidases [1, 8]. In addition to its role in NatA mediated cotranslational acetylation and propionylation [9], monomeric NAA10 is believed to have NatA independent roles, e.g. post-translational Nt-acetylation of proteins with neo-N-termini [10], acetylation of lysine side chains (K-acetylation) [11–13] and acetyltransferase independent roles such as protein-protein interactions with for instance DNA methyltransferase 1 (DNMT1) or p21-activated kinase-interacting exchange factor (PIX) [14–16]. *NAA10* is an essential gene in *Trypanosoma brucei*, *Caenorhabditis elegans* and *Drosophila melanogaster* and essential for normal development in *Danio rerio* [17–20].

In the years since the human NatA complex was identified and characterized, both *NAA10* and *NAA15* have repeatedly been associated with different types of cancer, suggesting a role in regulating cell proliferation and survival [21]. In recent years several different *NAA10* variants have been linked to rare genetic disorders. In 2011, a *NAA10* c.109 T > C p.(S37P) missense variant was identified as the cause of the X-linked Ogden syndrome (the locus for *NAA10* is Xq28) [22–24]. The patients affected by Ogden syndrome were all boys from two independent families (5 in one, 3 in the other). Ogden syndrome is associated with severe developmental delay (DD), a lipodystrophic facial appearance, short stature, microcephaly, cardiac arrhythmias and multiple malformations. All of the boys died before 2 years of age. Carrier females of the Ogden syndrome NAA10 variant all have skewed (> 90%) X-inactivation patterns and have no reported phenotypes [23]. Casey and colleagues described a second *NAA10* missense variant, c.128A > C, p.(Y43S), in two adult male patients with syndromic intellectual disability (ID), cardiac dysfunction (long-QT interval) and scoliosis, but not Ogden syndrome [25]. The variant was de novo in their mildly affected mother; a female with learning problems and heart disease (long-QT interval and ventricular tachycardia). Her blood leukocyte X-inactivation pattern was balanced.

In addition to the two missense variants described above, several *NAA10* variants that also affect females have been identified. Esmailpour and colleagues identified a 2 bp splice donor site deletion (c.471 + 2 T > A) in three brothers and an uncle with Lenz micropthalmia syndrome [26], a condition with ID, dysmorphic features and other malformations. Heterozygous carrier females may have mild manifestations [26]. Another *NAA10* variant, c.346C > T p.(R116W) was identified both in a female and a male, the latter being more severely affected [27]. In addition four *NAA10* missense variants c.319G > T p.(V107F), c.247C > T p.(R83C), c.382 T > A p.(F128I) and c.384 T > A p.(F128 L) have been found in female patients with moderate, severe or profound ID, postnatal growth failure, as well as skeletal and cardiac anomalies [27, 28].

Here we describe an 11 year old female with mild/moderate non-syndromic ID. Whole exome sequencing (WES) revealed de novo occurrence of a previously undescribed *NAA10* missense variant c.332 T > G p.(V111G). Functional testing demonstrated a decreased stability of overexpressed NAA10-V111G, decreased monomeric NAA10-V111G catalytic activity, while the NatA catalytic activity remained unchanged.

## Case presentation

### Patient description

The patient is a girl, now 11 years old, born three weeks preterm by section due to transverse lie, birth weight 2720 g. There were no feeding difficulties in infancy or later. Motor and language development was delayed: she walked without support at age 2½ years, and at age 3 years she said her first words. At age 10 years she knew the alphabet and tried to put letters together. She used diapers until age 8–9 years. Her sleep pattern is mildly irregular with frequent awakenings. Congenital malformations or epilepsy have never been detected. She has normal stature with length along the 25th–50th centile and weight along the 25th centile, but her head circumference has been in the lower normal range (2.5th - 5th centile). Her facial features are also normal - she is not clearly dysmorphic. Behavior is normal without autistic traits, but bruxism is a problem. She prefers the company of younger children. Her intellectual level is judged to be comparable to mild-moderate ID, formal IQ testing has not yet been done. At age 9 years trio whole exome sequencing (WES), comparing child to parental DNA sequences, revealed a de novo *NAA10* (NM_003491.3) c.332 T > G p.V111G variant which was further confirmed by Sanger sequencing.

### NatA homology model, prediction tools, structural conservation.

**Fig. 1:**
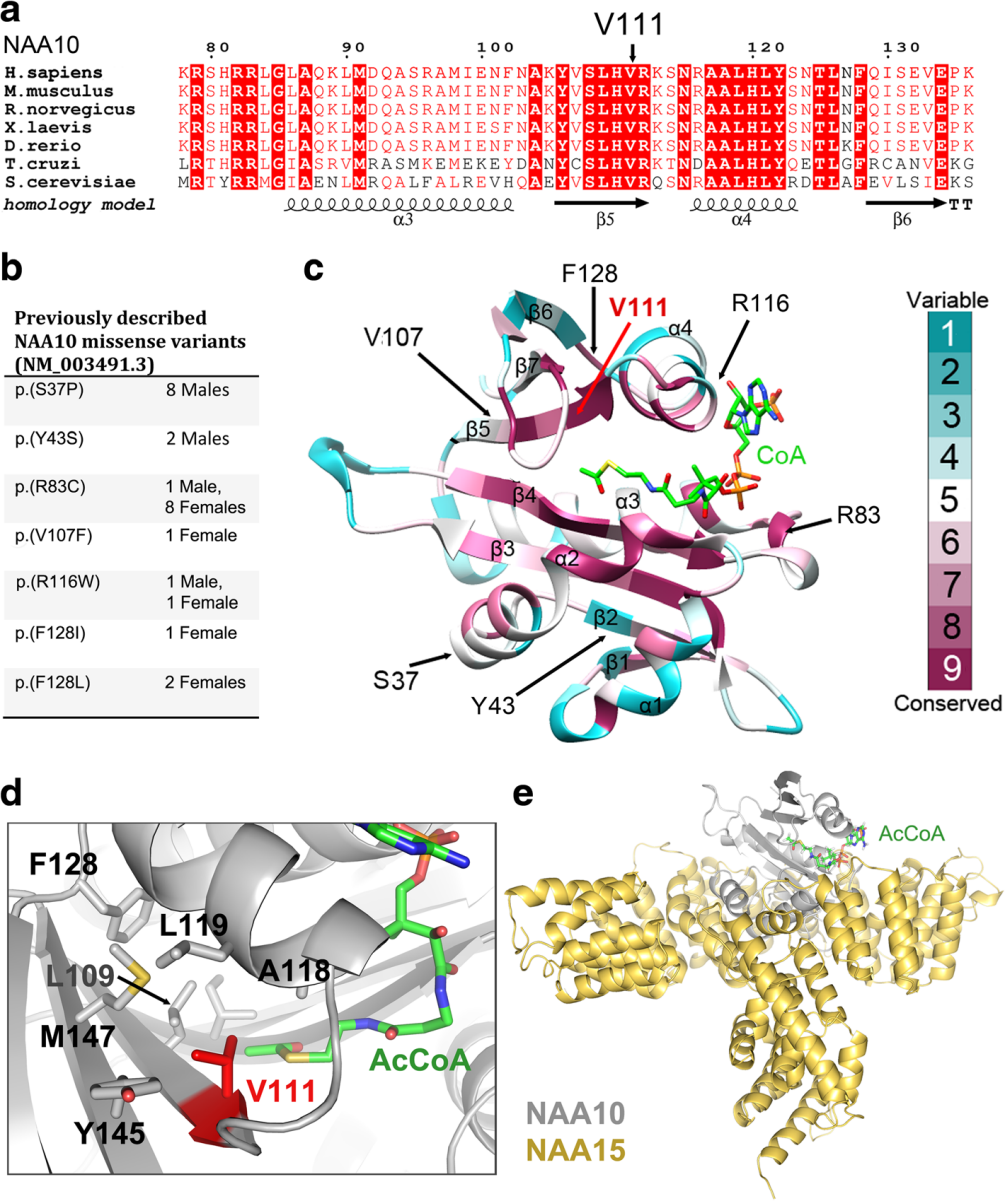
NAA10 multiple sequence alignment and structural conservation. **a** NAA10 multiple sequence alignment showing amino acids 78–137 (of human NAA10) (**b**) Previously described NAA10 variants identified in patients with ID/DD. **c** Cartoon representation of NAA10 colored with respect to evolutionary conservation. More conserved regions are colored in dark magenta, less conserved regions are colored in dark cyan. V111 is located towards the end of β5 and is highly conserved throughout evolution. **d** The side chain of V111 is pointing towards a hydrophobic pocket together with Y145, M147, L109 and L119. V111 is also located in close proximity to the sulfur-acetyl group of AcCoA, and to the β6-β7 loop region that is very important for peptide substrate binding. **e** Homology model of the human NatA complex. The auxiliary subunit NAA15 is shown in yellow cartoon, the catalytic subunit NAA10 is shown in white cartoon and the AcCoA is shown as green sticks

In line with other *NAA10* missense variants, the V111G substitution affects a highly conserved amino acid (Fig. 1, panels A and B). NAA10 is a 235 amino acid protein which adapts the characteristic GNAT fold common to NAT catalytic subunits. This fold consists of six or seven β strands and four α helices (Fig. 1c). β-strands 2-5 constitute the core of the protein. These four β strands, together with the α2 helix and the β6-β7 loop important for substrate binding, are highly conserved. V111 is located towards the end of the β5 strand, and a valine in this position is highly conserved in NAA10 homologues as well as in several other NAT catalytic subunits for which crystal structures have been solved (Fig. 1c, data not shown (PDB ID: 5K04 (NAA20), 4U9W (NAA40), 3FTY (NAA50), 5ICV (NAA60) and 4LX9 (ssNAT)) [29–33]. The side chain of V111 is forming a hydrophobic pocket together with Y145, M147, L119 and L109. It is also in close proximity (3.7 Å) to the sulphur group of Acetyl-CoA (AcCoA), which could indicate a role for V111 in positioning of AcCoA (Fig. 1d). A glycine in this position will not cause any steric clashes, but loss of the more bulky hydrophobic side chain of valine may possibly cause structural alterations affecting protein stability or AcCoA binding.

### Functional testing

**Fig. 2:**
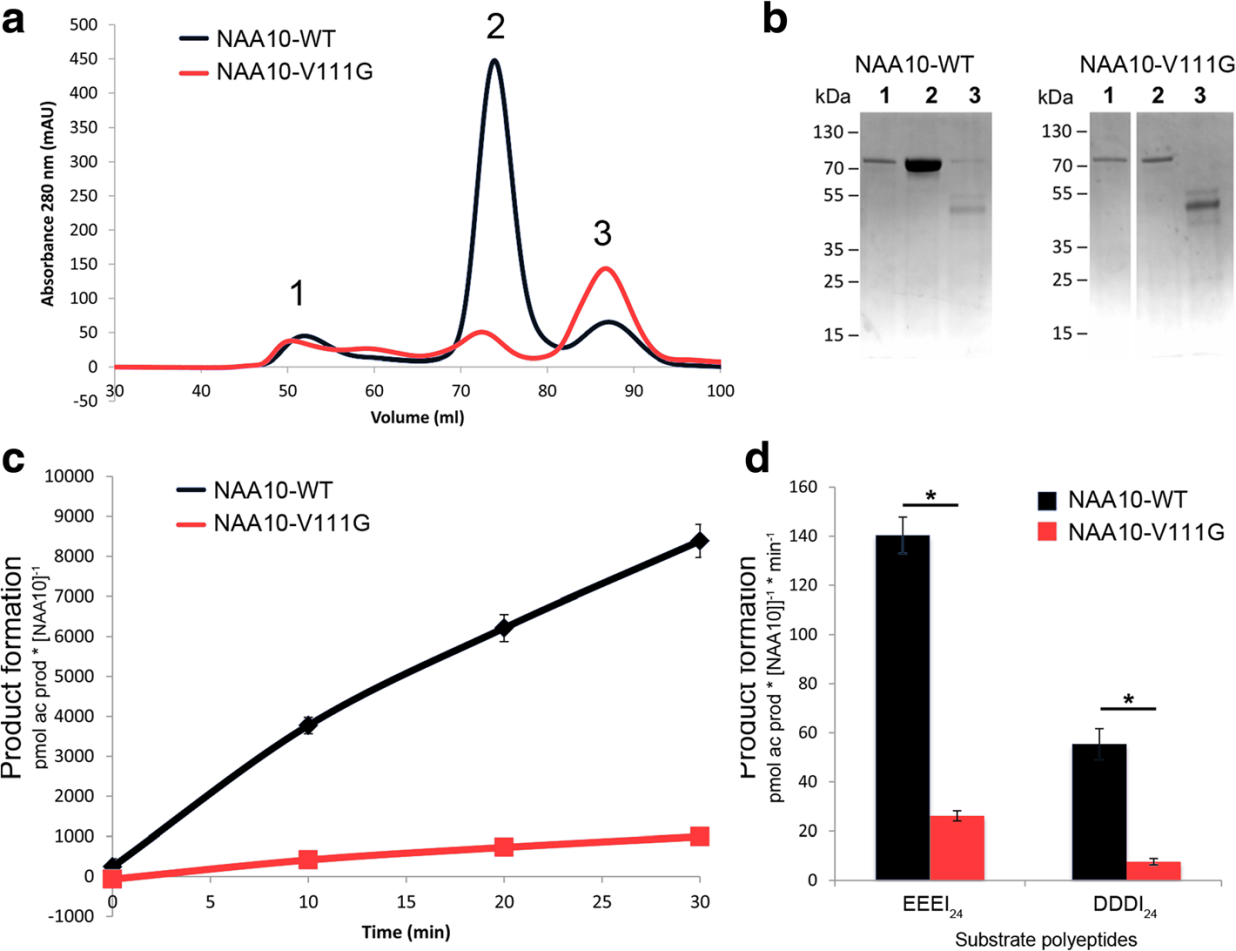
Protein purification and in vitro N-terminal acetyltransferase assays (**a**). Size exclusion chromatography of His/MBP-NAA10-V111G and His/MBP-NAA10-WT. Both the void volume (peak 1), and the monomeric peak at approximately 74 ml (peak 2) contain His/MBP-NAA10-WT or His/MBP-NAA10-V111G. The peaks corresponding to monomeric NAA10 (approximately at 75 mL) was further used for enzymatic experiments. **b** All fractions were analyzed by SDS-PAGE. **c** Time dependent Nt-acetylation of the substrate peptide EEEI_24_ catalyzed by His/MBP-NAA10-WT and His/MBP-NAA10-V111G. **d** Nt-acetylation of substrate peptides EEEI_24_ and DDDI_24_ catalyzed by His/MBP-NAA10-WT and His/MBP-NAA10-V111G. * statistically significant (*p* < 0.0001, student t-test)

In order to functionally assess NAA10-V111G, we first expressed His/MBP-NAA10-WT and His/MBP-NAA10-V111G in *E.coli*, purified enzymes and tested the in vitro NAT activity. Contrary to His/MBP-NAA10-WT, it was difficult to obtain good protein expression of His/MBP-NAA10-V111G, and a substantial fraction of the purified His/MBP-NAA10-V111G molecules eluted in the void volume of the size exclusion chromatography column (Fig. 2, panels A and B). This indicate that parts of the protein aggregate in units larger than 200 kDa, most likely due to an alteration of the protein structure, or reduced protein stability. Enzymes that eluted at a column volume corresponding to monomeric His/MBP-NAA10-V111G were tested for catalytic activity and shown to have an approximately 85% reduction in catalytic activity compared to His/MBP-NAA10-WT (Fig. 2, panels C and D).

**Fig. 3:**
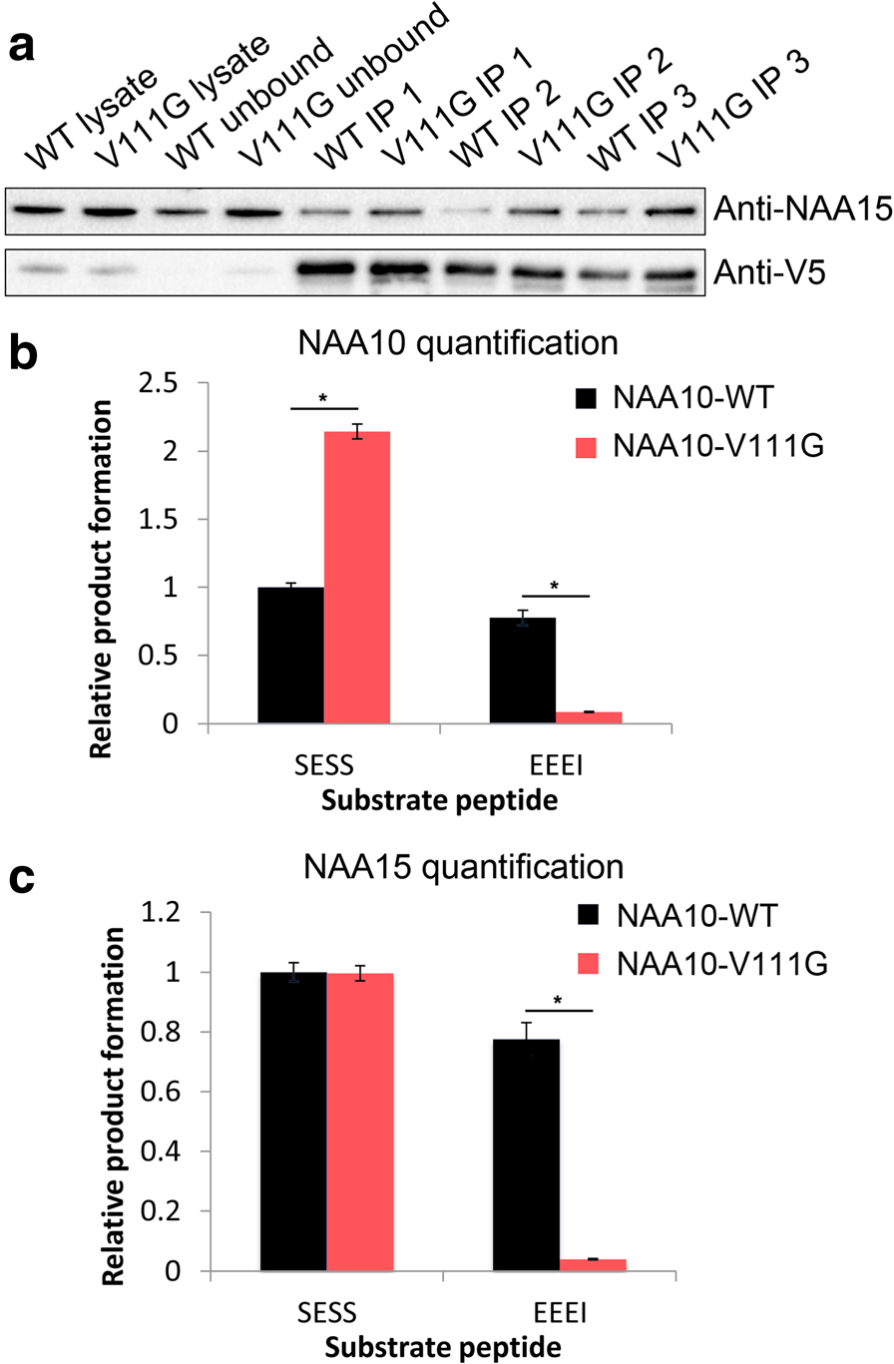
NAT-activity of immunoprecipitated NAA10-WT-V5 and NAA10-V111G-V5. **a** Western blot of immunoprecipitated NAA10-WT-V5 and NAA10-V111G-V5 using anti-V5. **b** Catalytic activity of immunoprecipitated NAA10-WT-V5 and NAA10-V111G-V5 correlated towards the amount of NAA10-V5 present in each sample. **c** Catalytic activity of immunoprecipitated NAA10-WT-V5 and NAA10-V111G-V5 correlated towards the amount of NAA15 present in each sample. NAT activity experiments were performed in triplicates. * statistically significant (*p* < 0.0001, student t-test). The data presented are representative for three independent experiments

Due to the low expression levels and protein yield of NAA10-V111G from *E.coli* transfection and protein purification, we transfected HeLa cells with plasmids coding for either V5-tagged NAA10-WT or V5-tagged NAA10-V111G followed by immunoprecipitation (IP) of the overexpressed protein by an anti-V5 antibody. Thereafter, NAT activity in the precipitate was measured (Fig. 3). NAA10 and NAA15 form a high-affinity protein complex (Fig. 1e), and as expected endogenous NAA15 co-immunoprecipitated with both overexpressed NAA10-WT-V5 and NAA10-V111G-V5. The amount of NAA10 and NAA15 present in each sample were determined by SDS-PAGE and Western blotting. Bands from the Western blot were quantified, and the measured catalytic activity was correlated with the amount of NAA10-V5 present in the sample (i.e. a mixture of monomeric NAA10 and NAA10 in complex with NAA15 – the NatA complex), and separately correlated with the amount of NAA15 present in the sample (i.e. the amount of the NatA complex only). Results from the IP-activity experiment corresponded well with our previous finding (Fig. 2): the ability of NAA10-V111G to acetylate the acidic N-termini EEEI_24_ was greatly reduced (Fig. 3, panels B and C). However, it also revealed a second interesting feature: As can be seen from the Western blot in Fig. 3a, more NAA15 co-immunoprecipitated with NAA10-V111G-V5 compared to NAA10-WT-V5. This was also reflected in our NAT-activity measurements where the immunoprecipitated NAA10-V111G-V5 sample showed higher NatA product formation (for the NatA substrate polypeptide SESS_24_) compared to the immunoprecipitated NAA10-WT-V5 sample when correlating for the amount of total NAA10-V5 present in the sample (Fig. 3b). However, when correlating for the amount of NAA15 present in each sample, the NatA product formation per NAA15 molecule (and thus NatA complex) was approximately equal (Fig. 3c). As monomeric NAA10 has a 1000-fold lower NAT-activity towards NatA substrates compared to the NatA complex [10], the contribution of monomeric NAA10 on acetylation of SESS_24_ is minimal. Taken together, this suggest that NAA10-V111G has reduced catalytic activity in a monomeric form, but not in complex with NAA15.

### NAA10-WT and NAA10-V111G protein turnover

**Fig. 4:**
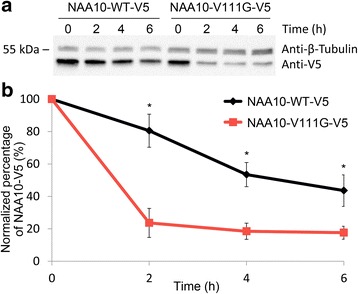
Cycloheximide chase experiments. **a** Western blot showing the protein levels of overexpressed NAA10-WT-V5 and NAA10-V111G-V5 before and 2, 4 and 6 h after protein synthesis was stopped by the addition of cycloheximide. **b** Quantification of NAA10-V5 bands from cycloheximide chase experiments. Western blots from 6 independent experiments were quantified using Imagelab 3.0 from Biorad, and the intensities in each band 2, 4 and 6 h after cycloheximide treatment were correlated both to the loading control (anti-β-tubulin) and to the intensity in the band representing NAA10-V5 before cycloheximide treatment. Each curve represents the average levels of NAA10 from 6 independent experiments combined. * statistically significant (*p* < 0.0005, student t-test)

In order to assess protein turnover of NAA10-WT and NAA10-V111G, we expressed V5-tagged NAA10-WT and NAA10-V111G in HeLa cells and performed cycloheximide-chase experiments. As can be seen from Fig. 4, NAA10-V111G-V5 has a higher turnover rate than NAA10-WT-V5. 2 h after cycloheximide treatment, the average amount of NAA10-V111G-V5 was reduced by approximately 80%, while NAA10-WT-V5 molecules was reduced by 20% relative to the amount of NAA10 molecules before cycloheximide treatment. Although the amount of NAA10-V111G-V5 is drastically decreased after 2 h, the level of NAA10-V111G-V5 seems to stabilize at around 20%. Most likely overexpressed NAA10-V111G-V5 is present both in an unstable monomeric form that is rapidly degraded and in complex with NAA15, which stabilizes the enzyme and protects it from degradation.

## Methods

### Trio exome sequencing

Whole exome sequencing was performed on trios on genomic DNA isolated from blood. DNA samples were prepared using the SeqCap EZ MedExome Kit (Roche, Bazel, Switzerland) and followed by paired-end 150 nt sequencing on the Illumina NextSeq500. Alignment and variant calling was performed as previously described [34]. Average median coverage of the target region was 207X with 100% of target region covered with at least 20 reads. Data annotation and interpretation were performed using the Cartagenia Bench Lab, NGS module (Cartagenia, Leuven, Belgium). The *NAA10* variant was verified by targeted Sanger sequencing. Informed consent was obtained from patient index and family members.

### Multiple sequence alignment, conservation scores and homology model.

Multiple sequence alignments were created using ClustalX [35] and the illustration using ESPript3.0 [36]. Conservation scores, and coloring schemes for the NAA10 model shown in Fig. 1b was made using ConSurf [37–40]. The human NatA homology model was created and published previously [23].

### Preparation of plasmids

In order to study the NM_003491.3 c.332 T > G (p.V111G) missense variant a bacterial expression vector pETM-41/His-MBP-*NAA10* and mammalian expression vector pcDNA3.1/*NAA10-*V5-His were modified by site-directed mutagenesis (Q5**®** Site Directed Mutagenesis Kit, New England Biolabs) according to the manufacturer’s protocol.

### Protein expression in *E.coli* BL21 cells

In order to study the in vitro catalytic activity of the novel NAA10 variant, recombinant His/MBP-NAA10-WT and His/MBP-NAA10-V111G were expressed in BL21 Star™ DE3 competent *E. coli* cells for protein purification. Cells were grown to an OD_600_ of 0.6 and protein expression was induced by adding 0.5 mM IPTG to the LB-media. The culture was incubated overnight at 18 °C to allow sufficient expression of His/MBP-NAA10. The following morning, the culture was centrifuged at 4750 rpm for 20 min to harvest cells and cell pellets were stored at − 20 °C until use. A two-step purification protocol (affinity chromatography and size exclusion chromatography was performed as described [28]) and the purified protein was analysed by SDS-PAGE and coomassie.

### In vitro acetylation assays

In vitro DTNB-based acetylation assays were performed to study the catalytic activity of the purified His/MBP-NAA10 variants as described [27]. In short, acetylation assays were performed simultaneously for both His/MBP-NAA10 WT and His/MBP-NAA10 V111G in order to compare their catalytic activity. 500 μM substrate polypeptide, 500 μM acetyl-CoA, 1× Ac buffer, ddH_2_0 and enzyme were mixed to a final volume of 50 μl. In addition, two negative replicates without enzyme were prepared. Samples were incubated at 37 °C on a heating block and the reaction was quenched at different time points by adding 100 μl Quenching buffer (3.2 M guanidine-HCl, 100 mM Na_2_HPO_4_ pH 6.8). Samples were then mixed with 20 μl of DTNB buffer (100 mM Na_2_HPO_4_, 10 mM EDTA pH 6.8) saturated with DTNB, and the absorbance was measured at 412 nm using an Epoch microplate reader.

### Immunoprecipitation of NAA10-WT-V5 and NAA10-V111G-V5

For each IP experiment, five 10 cm dishes of HeLa cells (ATCC, CCL-2) were transfected with 4 μg NAA10 WT-V5 and eight dishes were transfected with 10 μg of each NAA10**-**V111G-V5. 6 μg of empty V5-vector was co-transfected with NAA10-WT-V5 to ensure equal conditions for the cells. 48 h post transfection, cells were harvested using a cell scraper, and centrifuged at 1500 *x g* for 5 min. The resulting cell pellet was washed in 5 ml 1× PBS, centrifuged again and lysed in 350 μl IPH buffer containing 1× protease inhibitor per cell dish on ice for 30 min. The lysed cells were then centrifuged at 17000 *x g* for 1 min to remove cell debris and the supernatant was transferred to a new tube. 30 μl cell lysate was saved for Western blot analysis.

Cell lysates were incubated with 5 μg anti-V5 on a rotating wheel at 4 °C for 2–3 h to allow formation of immune complexes. 60 μl washed magnetic beads were then added to each IP sample and incubated at 4 °C on a rotating wheel overnight to retrieve the immune complexes. The next day, the IP samples were placed on a magnetic holder and 30 μl of the supernatant was saved for western blot analysis. The IP beads were washed 3× in 1 ml IPH buffer and 2× in acetylation buffer. Finally the washed IP beads were resuspended in 95 μl acetylation buffer and were ready to be used in C14 acetylation assays.

### Carbon-14 acetylation assay

C14 acetylation assays were performed to assess the intrinsic NatA activity of the immunoprecipitated NatA complexes. Acetylation assays were performed simultaneously for mutant and WT IP samples in order to compare their catalytic activity. Three positive replicates of 200 μM specific peptide (SESS_24_ or EEEI_24_), 100 μM unlabeled AcCoA, 100 μM 14C-labelled AcCoA, 10 μl IP beads and dH_2_O were mixed to a final volume of 25 μl. In addition, two negative replicates without peptide were prepared. The samples were incubated at 37 °C and 1300 rpm on a thermo shaker for 35 min. After incubation, the samples were placed on a magnet holder to isolate the magnetic beads and 23 μl sample was transferred to P81 phosphocellulose discs. The paper discs were then washed 3 × 5 min in 10 mM HEPES buffer followed by air drying on paper. The dried discs were placed in individual tubes, filled with 5 ml scintillation fluid and the C14 signal was measured using a scintillation counter. The IP samples were analyzed by Western blotting (as briefly described above) and the measured activity was adjusted to quantification of corresponding anti-V5 bands.

### Cycloheximide chase experiments

HeLa cells were cultured in DMEM growth medium (Sigma) supplemented with 10% fetal bovine serum (FBS) and 3% L-Glutamine and incubated at 37 °C with 5% CO_2_. Cells were transfected at approximately 60–70% confluence, in the log-phase of growth, to maximize the transfection efficiency. 3 × 4 wells of cells in 6-well plates were prepared per NAA10 variant (WT and V111G). Cells were transfected with 2.6 μg of V5-vector encoding NAA10 WT or V111G using XtremeGENE 9 DNA transfection reagent (Roche) according to the manufacturer’s protocol and DMEM growth medium was replaced 4–6 h post transfection. 48 h post transfection, the growth medium of each well was replaced with 2 ml medium containing 50 μg/ml Cycloheximide. Cells were harvested using a cell scraper at 0 h, 2 h, 4 h and 6 h after the cycloheximide addition. The cells harvested at time point 0 h were not treated with cycloheximide. Harvested cells were centrifuged at 4 °C using the “short spin” function of a Heraeus Fresco 17 centrifuge for 15 s with 17,000 g as the maximum speed and the cell pellets were washed in 1 ml cold 1× PBS, centrifuged again and stored at − 80 °C.

### Western blot analysis

In order to analyse NAA10 turnover, cell samples were analysed by Western blotting. Cell pellets were lysed by resuspension in 30 μl IPH lysis buffer (50 mM Tris-HCl pH 8.0, 150 mM NaCl, 5 mM EDTA and 0.5% NP-40, 1× complete EDTA free protease inhibitor) and incubation on ice for 20 min. Cell debris was removed by centrifugation at 4 °C and 17,000 *x g* for 8 min and the supernatant was analysed by SDS-PAGE and Western blotting as described [25]. In order to visualize NAA10-V5 and β-tubulin, a 1:3000 dilution of primary ab (anti-V5, anti-tubulin) in 1% dry milk overnight at 4 °C with gentle shaking was used followed by a 1:3000 dilution of secondary ab anti-mouse or anti-rabbit (dependent on the primary ab) diluted in 3% dry milk for 1 h at room temperature with gentle shaking. HRP signals were developed using SuperSignal® West Pico Chemiluminescent Substrate (Thermo Scientific) followed by detection, imaging and quantification using ChemiDoc™ XRS+ connected with ImageLab™ v3.0 (Bio-Rad).

## Discussion and conclusions

In recent years the phenotypic spectrum associated with NatA-deficiency has greatly expanded. Several *NAA10* variants have been identified, some mainly affecting males [22, 25] and some affecting both males and females [27, 28]. Here we characterize a previously undescribed NAA10 de novo missense change, p.(V111G) in an 11 year old female with mild/moderate ID. We show that NAA10-V111G has a reduced stability and loss of catalytic activity in monomeric form, but most likely remains active and stable when bound to NAA15 in the NatA complex. V111 is positioned towards the end of the β5 strand, with a hydrophobic side chain contributing to a hydrophobic pocket in the core of the protein. Substituting the more bulky hydrophobic side chain of valine with a hydrogen atom only will cause loss of any hydrophobic contacts the valine side chain took part in. In addition, the unique achirality of glycine makes it able to adopt different Cα backbone rotation angles than all other amino acids, possibly increasing the flexibility in these bonds. Although V111 and the hydrophobic pockets surrounding V111 is not in direct contact with NAA15 (Fig. 1e), our results indicate that the increased flexibility and/or reduced stability most likely is restored by NAA15 binding.

Several other *NAA10* missense variants have previously been found to affect NAA10 protein stability (NAA10-V107F [28], NAA10-Y43S [25], NAA10-F128I and NAA10-F128L [27]). With the exception of Y43, all of these residues are situated in one of the highly conserved β-strands β4, β5 or β6 in the core of the protein. The side chains of V107 and F128 (which were altered in other patients with ID) form a hydrophobic pocket in close proximity to the hydrophobic pocket surrounding V111 [27]. It is therefore conceivable that V111G cause disease through similar mechanisms as V107F, F128L and F128I variants. The phenotypic spectrum of this group of patients is very broad, ranging from learning problems (in a mother heterozygous for Y43S) to severe intellectual disability, heart disease (arrhythmias) and short stature. Females can also have a severe phenotype as illustrated by three females with de novo V107F, F128I and F128L variants, respectively [27, 28]. In contrast, female carriers of the p.(S37P) variant causing Ogden syndrome appear to be normal [23], probably because the missense variant is severe enough to skew X-inactivation at an early stage of development. Saunier and colleagues described seven unrelated females sharing the NAA10-R83C missense change. In five of them, the blood leukocyte X-inactivation patterns were determined. It was normal (balanced) in three, and skewed (92% and 100%) in two. The girl with 100% skewing was the only NAA10-R83C individual without microcephaly or postnatal growth retardation [27]. X-inactivation patterns were also tested in four of the female patients with NAA10 variants affecting the same hydrophobic pocket as V111 [27, 28]. All had random X-inactivation patterns, in line with lack of phenotype rescue by early deselection of cells expressing the *NAA10* variants. Our patient had an X-inactivation pattern of 80/20, phase unknown, which is within our defined normal range of 15–85%.

Although a lot is known about NAA10 function when in complex with NAA15, the functions of monomeric NAA10 remain disputed. Although an in vivo NAT-substrate for monomeric NAA10 is yet to be found, it has a strong substrate preference for acidic N-termini in vitro and should be capable of catalyzing post-translational Nt-acetylation of neo-N-termini [10]. Monomeric NAA10 has also been described both as a lysine acetyltransferase (KAT) [11–13] and to excerpt functions by stably interacting with other proteins [14–16], although some of these reports remain somewhat controversial. An early finding that NAA10 is acting as a negative regulator of the transcription factor HIF1α through lysine acetylation [41] was for instance contradicted by several reports in the following years [42–44] and more recently Magin and colleagues showed that purified NAA10 exhibits undetectable KAT activity in their assays [45]. In depth studies of the NAA10-S37P causing Ogden syndrome revealed that NAA10 had a reduced capability to form a functional NatA complex, that it had a reduced monomeric NAT activity, and proteomic studies of primary cell lines revealed a reduced level of Nt-acetylation of NatA substrates mainly [23, 24]. It is likely that loss of NatA function is more crucial than loss of monomeric NAA10 function due to the thousands of cellular NatA substrates. Our data suggest that the altered structure and reduced stability of NAA10-V111G mainly affect monomeric NAA10 function, and further that this may be a common feature for several previously described cases of pathogenic NAA10 variants. Defining the exact disease mechanism in cases like Ogden syndrome and other NAA10 related disorders is challenging, and it is not unlikely that it has a pleiotropic nature involving multiple substrates and signaling pathways. More studies are thus needed in order to understand whether NAA10 p.(V111G) (and other pathogenic NAA10 variants) are causing disease due to loss of N-terminal acetylation, loss of K-acetylation, loss of N-acetyltransferase independent functions or through dominant negative effects.

## Abbreviations

AcCoA: Acetyl Coenzyme A
DD: Development delay
DNMT1: DNA methyltransferase 1
ID: Intellectual disability
IP: Immunoprecipitation.
NAA10: N-alpha acetyltransferase 10
NAA15: N-alpha acetyltransferase 15
NAT: N-terminal acetyltransferase
NatA: N-terminal acetyltransferase A
PIX: p21-activated kinase-interacting exchange factor
WES: Whole exome sequencing
